# Modulating the immune response to reduce hypertension-associated cardiovascular damage

**DOI:** 10.1172/JCI158280

**Published:** 2022-03-15

**Authors:** Daniel Henrion

**Affiliations:** Angers University, MitoVasc Department, Team 2 (CarMe), Angers University Hospital (CHU of Angers), CNRS, INSERM, Angers, France.

## Abstract

Cardiovascular diseases are a leading cause of mortality and disability worldwide. Hypertension, a major risk factor for these diseases, remains difficult to treat despite numerous drugs being available. In this issue of the *JCI*, Failer et al. show that the endogenous antiinflammatory agent developmental endothelial locus-1 (DEL-1) decreased blood pressure and cardiac and aortic hypertrophy in mouse models of hypertension through reduction in **α**_v_**β**_3_ integrin–dependent metalloproteinase activity and immune cell recruitment, leading to reduced production of proinflammatory cytokines in cardiovascular tissues. This study offers an alternative in the treatment of hypertension-mediated organ damage through the immunomodulatory effect of DEL-1.

## Avenues for targeting hypertension

The endothelium plays a central role in the control of vascular tone through the production and release of numerous vasoactive agents, among which nitric oxide (NO) is the best known. In healthy subjects, the endothelium has also antithrombotic, antiproliferative, and antiinflammatory properties (1). The ability of the endothelium to maintain vascular equilibrium is reduced by risk factors such as aging, smoking, diabetes, obesity, and hypertension (1, 2). Hypertension remains the most common modifiable risk factor for cardio- and cerebrovascular disorders and death. The most frequently used treatments are the angiotensin I–converting enzyme inhibitors and the angiotensin II type 1 receptor blockers, sometimes in combination with calcium channel blockers or diuretics (3). Although numerous antihypertensive drugs are available, a large proportion of patients remain with excessive blood pressure and consequently with a high risk of long-term morbidity. Thus, the search for alternative treatments remains essential to overcoming this gap. Drugs targeting the renin-angiotensin-aldosterone system with, for example, antialdosterone agents or aldosterone synthase inhibitors, angiotensin-converting enzyme 2 (ACE2) activators, or MAS agonists are under development in addition to aminopeptidase inhibitors acting centrally, vasopeptidase inhibitors, inhibitors of the intestinal Na^+^/H^+^ exchanger 3, and vasoactive intestinal peptide receptor blockers, among others (4). Vaccines are also undergoing evaluation to address the main limitation of antihypertensive therapies, compliance with treatment. Thus, vaccines offer an opportunity, as patients would have no more pills to take, or to forget, every day (5). Vaccines targeting the renin-angiotensin system have been tested for over 50 years with some success in lowering blood pressure in animals, but with too heavy side effects for common use. More recent approaches allow vaccines to target angiotensin II or its type 1 receptor (4, 5). Interestingly, hypertension is associated with inflammation (6, 7), and targeting the immune system directly is another promising therapeutic avenue that is now being investigated in depth (6).

## Modulation of the immune system

**Figure 1:**
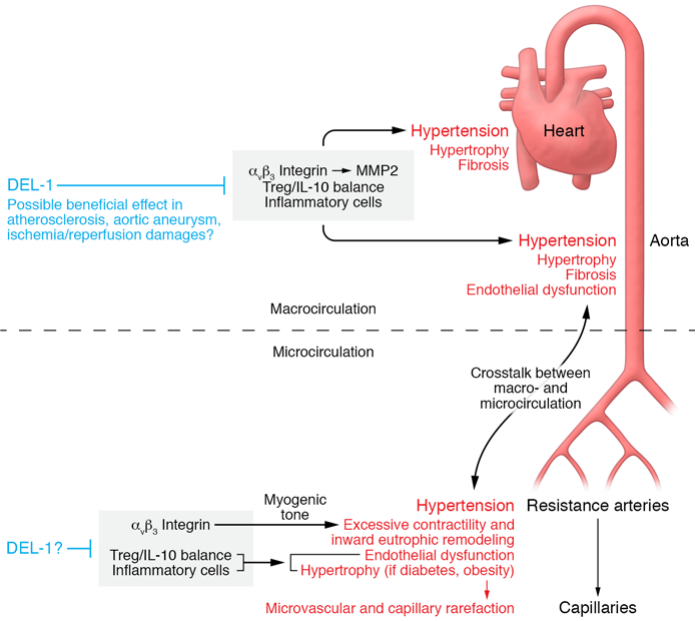
DEL-1 protects against hypertension-induced cardiovascular damage. Failer et al. (8) demonstrated that the immunomodulator protein DEL-1 reduced α_v_β_3_ integrin–mediated activation of MMP2, leading to decreased inflammation in the heart and in the aorta in hypertension. This protection involved an improvement of the Treg/IL-10 balance and a reduction in immune cell infiltration. The crosstalk between macro- and microcirculation induces a vicious cycle in hypertension, whereas an efficient treatment affecting both could lead to a virtuous circle.

Failer et al. (8) offer a tool for the treatment of hypertension and especially the treatment of the vascular and cardiac hypertrophy associated with hypertension, cardiac failure, and death. Developmental endothelial locus-1 (DEL-1) is an endogenous antiinflammatory glycoprotein that reduces immune cell recruitment (9). Notably, DEL-1 could fill a void in the treatment of hypertension between pharmacological tools and vaccines. The authors showed that DEL-1 stopped the increase in blood pressure and normalized endothelium-dependent relaxation in two different mouse models of hypertension: one with angiotensin II infusion and one with volume overload and thus reduced renin-angiotensin activity (Figure 1). The findings suggest that DEL-1 could efficiently prevent both essential and secondary hypertension. Indeed, in hypertensive mice, both the endothelial overexpression of DEL-1 and the injection of recombinant DEL-1 suppressed aorta and heart hypertrophy and fibrosis and improved left-ventricular function and coronary perfusion. Thus, DEL-1 seems efficient against the main deleterious consequences of hypertension and offers an opportunity for the management of hypertension through modulation of the immune system.

Whereas the study by Failer et al. (8) shows an efficient effect of DEL-1 on cardiac and aortic hypertrophy, hypertension involves a complex crosstalk between macrocirculation and microcirculation (Figure 1). As described in a recent review, “The two are tightly interconnected into a dangerous cross-link during hypertension” (3). The effect of DEL-1 on microcirculation remains to be explored, and the possible role of DEL-1 in the structure and function of resistance arteries is an important topic for future research. Indeed, these small arteries have a major role in blood pressure control and in patients with hypertension resistance arteries undergoing inward eutrophic remodeling (3). Although resistance artery remodeling excludes substantial changes in the amount of wall tissue and generally maintains the media cross-sectional area, increased oxidative stress and inflammation have been demonstrated in hypertension (10). Nevertheless, hypertrophic remodeling has been observed in resistance arteries in hypertensive people with diabetes and obesity and in several forms of secondary hypertension (11, 12). Thus, reducing inflammation using DEL-1 could also benefit small resistance artery remodeling, at least in hypertension associated with other risk factors. Additionally, the effect of DEL-1 on α_v_β_3_ integrins implies that DEL-1 could interact with resistance artery tone and hence affect inward arterial eutrophic remodeling observed in essential hypertension. Indeed, α_v_β_3_ integrins are involved in the myogenic constriction of resistance arteries (13). Excessive pressure-induced myogenic tone of these small arteries has a role in the development of hypertension and is a determinant of the eutrophic inward remodeling observed in essential hypertension (14–16).

DEL-1 is also efficient in promoting angiogenesis (17), an effect that could benefit hypertension-associated microvascular and capillary rarefaction (18). Thus, a treatment that increases DEL-1 activity may positively affect the microvascular rarefaction observed in hypertension, although DEL-1 had no obvious effect in peripheral arterial disease (19). DEL-1 has also been shown to inhibit postischemic revascularization in a mouse model of hind-limb ischemia (20). Thus, more investigation is needed to clarify this issue.

As DEL-1 reduces immune cell recruitment in the aorta, one can also postulate that it could be a useful tool against atherosclerosis and possibly aortic aneurysm. Indeed, chronic inflammation governs the progress of atherosclerotic lesions with a major role for adaptive immunity (21). Similarly, the inflammatory process plays a key role in abdominal aortic aneurysm with the involvement of different monocyte and macrophage subsets in the initiation and in the progression of the disease (22). Thus, future studies should focus on the role of DEL-1 in atherosclerosis and aortic aneurysm, as a modulator of the immune response with antiinflammatory properties is likely to have a therapeutic potential in these pathologies.

## Conclusions

Importantly, Failer et al. show that DEL-1 is beneficial in hypertension, acting through immunomodulatory and antiinflammatory effects. In the model of hypertension used by Failer et al. (8), the effects of DEL-1 were mediated by α_v_β_3_ integrin. The inhibition of α_v_β_3_ integrin by DEL-1 reduced pro-MMP2 activation and increased Treg numbers and IL-10 production, thus reducing the recruitment of inflammatory cells and proinflammatory cytokine production in cardiac and vascular tissues. Further investigation should assess the role of DEL-1 on the microcirculation and especially on resistance artery tone, which control blood pressure and blood flow to organs. Important experiments would also include testing DEL-1 on function and structure changes of resistance arteries and on the capillary rarefaction induced by hypertension. The findings by Failer and colleagues suggest that DEL-1 is likely to reduce atherosclerosis progression and prevent the development of aortic aneurysms and the damage following ischemia/reperfusion. Indeed, DEL-1 is likely to improve endothelium NO-dependent dilation and to reduce myogenic response in these small arteries. Thus DEL-1 could provide an antihypertensive tool alongside the existing therapeutic arsenal to fully protect patients with hypertension.

## Version 1 03/15/2022

Electronic publication
